# Single cell analysis reveals inhibition of angiogenesis attenuates the progression of heterotopic ossification in *Mkx*^*−/−*^ mice

**DOI:** 10.1038/s41413-021-00175-9

**Published:** 2022-01-07

**Authors:** Junxin Lin, Yuwei Yang, Wenyan Zhou, Chao Dai, Xiao Chen, Yuanhao Xie, Shan Han, Huanhuan Liu, Yejun Hu, Chenqi Tang, Varitsara Bunpetch, Dandan Zhang, Yishan Chen, Xiaohui Zou, Di Chen, Wanlu Liu, Hongwei Ouyang

**Affiliations:** 1 Dr. Li Dak Sum & Yip Yio Chin Center for Stem Cells and Regenerative Medicine, and Department of Orthopedic Surgery of the Second Affiliated Hospital, Zhejiang University School of Medicine, Hangzhou, China; 2 Department of Sports Medicine, Zhejiang University School of Medicine, Hangzhou, China; 3 Zhejiang University-University of Edinburgh Institute, Zhejiang University School of Medicine, and Key Laboratory of Tissue Engineering and Regenerative Medicine of Zhejiang Province, Hangzhou, China; 4 China Orthopedic Regenerative Medicine Group (CORMed), Hangzhou, China; 5 Department of Pathology, Zhejiang University School of Medicine, Hangzhou, China; 6 Clinical Research Center, The First Affiliated Hospital, School of Medicine, Zhejiang University, Hangzhou, China; 7 Center for Reproductive Medicine, The Second Affiliated Hospital, School of Medicine, Zhejiang University, Hangzhou, China

**Keywords:** Bone, Pathogenesis

## Abstract

Tendon heterotopic ossification (HO) is characterized by bone formation inside tendon tissue, which severely debilitates people in their daily life. Current therapies fail to promote functional tissue repair largely due to our limited understanding of HO pathogenesis. Here, we investigate the pathological mechanism and propose a potential treatment method for HO. Immunofluorescence assays showed that the Mohawk (MKX) expression level was decreased in human tendon HO tissue, coinciding with spontaneous HO and the upregulated expression of osteochondrogenic and angiogenic genes in the tendons of *Mkx*^*−/−*^ mice. Single-cell RNA sequencing analyses of wild-type and *Mkx*^*−/−*^ tendons identified three cell types and revealed the excessive activation of osteochondrogenic genes during the tenogenesis of *Mkx*^*−/−*^ tendon cells. Single-cell analysis revealed that the gene expression program of angiogenesis, which is strongly associated with bone formation, was activated in all cell types during HO. Moreover, inhibition of angiogenesis by the small-molecule inhibitor BIBF1120 attenuated bone formation and angiogenesis in the Achilles tendons of both *Mkx* mutant mice and a rat traumatic model of HO. These findings provide new insights into the cellular mechanisms of tendon HO and highlight the inhibition of angiogenesis with BIBF1120 as a potential treatment strategy for HO.

## Introduction

The tendon anchors muscle to bone and transmits the forces generated by musculoskeletal tissues to enable body movement. Increasing incident rates of tendon injuries due to strenuous exercise or age-related degeneration are becoming increasingly challenging for medical personnel. Tendon heterotopic ossification (HO), which is characterized by endochondral bone formation in tendons, is the major histological feature of tendon injury or tendinopathy at later stages^1,2^. However, the lack of knowledge about the pathogenesis of HO has hampered the development of effective therapies. Current clinical treatment options for HO are limited to physical therapy, the use of nonsteroidal anti-inflammatory drugs and surgical excision of the ectopically formed bone, which may cause frequent complications and a high recurrence rate^3^. Therefore, better understanding the cellular and molecular mechanisms of HO will provide new clues for novel therapeutic or prophylactic approaches.

Progenitor cells contributing to tendon HO have been evaluated by lineage tracing^4^. Recent studies have shown that Scleraxis (Scx)-expressing tendon progenitor cells contribute to HO in trauma or hyperactive bone morphogenetic protein (BMP) induced heterotopic ossification models. Constitutive expression of the active form of the type I BMP receptor ACVR1 in Scx-expressing cells results in a clear HO phenotype in mouse joints and tendons, even in the absence of injury. These pioneering studies indicate that the ectopic activation of osteogenic signaling confers osteogenic differentiation potential in tendon progenitor cells. However, various subpopulations of tendon resident cells exist^5–8^, and their contributions to the HO process has not been studied.

Mohawk (Mkx) is a tendon-specific transcription factor, and its mutation causes tendon HO in mice and rats^9–13^. Using *Mkx* knockout rodent models, recent studies revealed that tendon-derived cells exhibited increased expression levels of chondrogenic markers and accelerated chondrogenic and osteogenic differentiation during the progression of HO^13,14^. These studies provide critical information about the tendon HO phenotype and suggest that *Mkx* knockout animals can be employed as an appropriate model to study the cellular and molecular mechanisms governing the progression of HO.

In this study, we utilized *Mkx*^*−/−*^ mice as a model of tendon HO to explore its pathogenesis and potential treatments. Our results at the single-cell level suggest that tendon progenitor cells commit to an osteoblastic fate during HO progression. Moreover, tendon HO was shown to be associated with the upregulated expression of proangiogenic genes in all tendon cell clusters. Inhibition of angiogenesis by the small molecule BIBF1120 attenuated bone formation and angiogenesis in both the *Mkx*^*−/−*^ mouse degenerative HO model and the rat traumatic model of HO.

## Results

### MKX is suppressed in human HO, and *Mkx* knockout leads to HO in mouse tendons

**Fig. 1:**
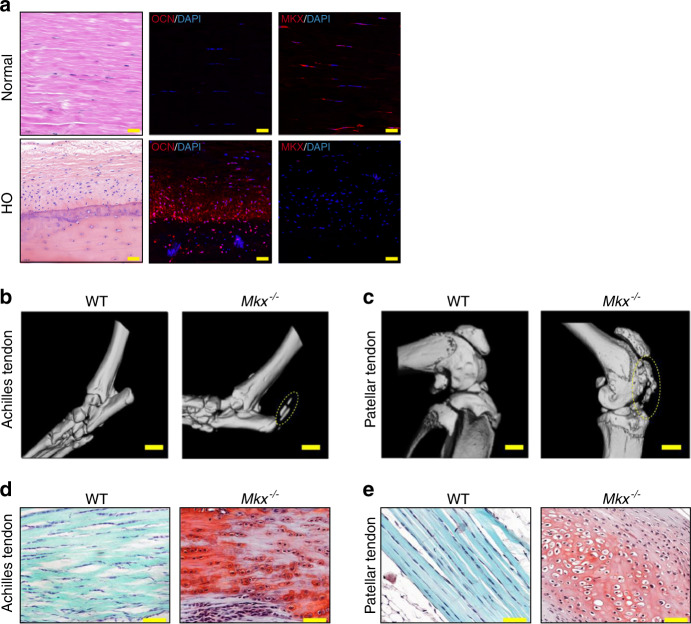
Mkx deficiency causes tendon heterotopic ossification. **a** HE (left) and immunofluorescence staining of OCN (middle) and MKX (right) in the tendons of normal individuals and HO patients. Scale bar, 50 μm. **b, c** Micro-CT examination of Achilles tendons (**b**) and patellar tendons (**c**) from WT and *Mkx*^*−/−*^ mice. Scale bar, 1 mm. **d, e** Safranin O-Fast green staining of Achilles tendons (**d**) and patellar tendons (**e**) from WT and *Mkx*^*−/−*^ mice. Scale bar, 50 μm

To assess the potential involvement of MKX in human HO pathogenesis, we evaluated the expression of MKX in the tendons of normal individuals and HO patient by immunofluorescence analysis. Hematoxylin and eosin (H&E) staining revealed HO in human tendons with clear tendon layers, well-developed bone and their interface. Osteocalcin-positive (OCN^+^) osteoblasts were present at the interface and bone region. The MKX protein was detected in tendon cells from normal individuals (Fig. 1a). However, in the HO samples, the expression of MKX was dramatically decreased (Fig. 1a), indicating that MKX is involved in tendon HO in humans.

Previous studies reported HO in the Achilles tendons of *Mkx*-deficient rats and mice^13,14^, but whether tendons from other sites are affected and how HO develops remain unknown. To further characterize the tendon heterotopic ossification phenotype, we analyzed wild-type (WT) and *Mkx*^−^^*/*^^−^ mice by microcomputed tomography (micro-CT) and histological staining. As expected, ectopic bone was clearly observed in the Achilles tendons of *Mkx*^*−/−*^ mice, confirming previously reported HO phenotype (dotted circles in Fig. 1b)^14^. Importantly, the *Mkx*^*−/−*^ mice also exhibited a clear and detectable heterotopic bone formation phenotype in their patellar and tail tendons at 8 weeks of age (dotted circles in Fig. 1c and Supplementary Fig. S1). To further confirm the HO phenotype in *Mkx*^*−/*−^ tendons, we performed safranin O and fast green (SOFG) staining. In WT Achilles and patellar tendons, clear elongated nuclei labeled by dark blue staining lined the extracellular matrix (stained a light blue), which is a characteristic of tendon architecture (Fig. 1d, e). In contrast, abundant proteoglycans, indicated by red staining, and chondrocytes with rounded nuclei were observed in the Achilles and patellar tendons of *Mkx* mutants, confirming the characteristic chondral lesions and endochondral ossification observed in HO (Fig. 1d, e). These observations confirm that *Mkx* deficiency leads to HO in both Achilles, patellar and tail tendons. Taken together, these results indicate that *Mkx* knockout mice represent a suitable model for elucidating the pathogenic mechanisms of tendon HO.

### Upregulation of osteochondrogenic and angiogenic genes in HO tendons

**Fig. 2:**
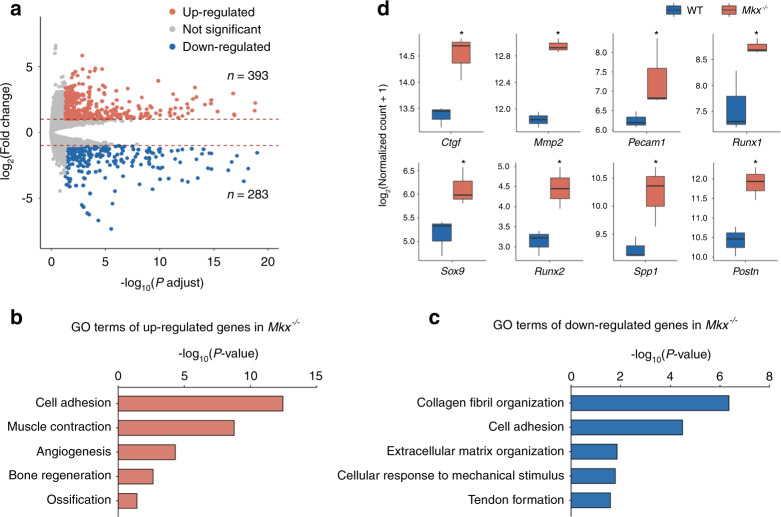
Transcriptomic analysis of WT and *Mkx*^*−/−*^ tendons. **a** Volcano plot of differentially expressed genes between WT and *Mkx*^*−/−*^ tendons. **b** Representative enriched GO terms corresponding to upregulated genes in *Mkx*^*−/−*^ tendons. **c** Representative enriched GO terms corresponding to downregulated genes in *Mkx*^*−/−*^ tendons. **d** Boxplots of the expression levels of genes related to angiogenesis (*Ctgf*, *Mmp2*, *Pecam1* and *Runx1*) and osteochondrogenesis (*Sox9*, *Runx2*, *Spp1* and *Postn*) in WT and *Mkx*^*−/−*^ tendons. *:*p* < 0.05

To investigate the molecular mechanism of tendon HO, we first performed bulk RNA sequencing (RNA-seq) on tail tendons isolated from 4-week-old *Mkx*^*−/−*^ and WT mice. We then analyzed differentially expressed genes (DEGs) between *Mkx*^*−/−*^ HO and WT control tissues to identify genes that are potentially involved in HO (using cutoff thresholds of a fold change greater than 2 and an FDR less than 0.05). DEG analysis revealed 393 upregulated genes and 283 downregulated genes in *Mkx*-deficient tendons (Fig. 2a). Gene ontology (GO) analysis suggested that the genes with upregulated expression in *Mkx*^*−/−*^ tendons were associated with cell adhesion, muscle contraction, angiogenesis, bone regeneration, and ossification (Fig. 2b), while genes with downregulated expression in *Mkx*^*−/−*^ tendons were enriched in collagen fibril organization, cell adhesion, extracellular matrix organization, response to mechanical stimuli, and tendon formation (Fig. 2c).

To identify potential signaling pathways regulated by Mkx, we performed Kyoto Encyclopedia of Genes and Genomes (KEGG) analysis of the genes that were upregulated upon *Mkx* knockout. The top 20 enriched pathways are shown in Supplementary Fig. S2a. Many genes were highly and specifically clustered in the Wnt, PI3K-Akt and Rap1 signaling pathways, which are importantly associated with the physiological and pathological features of angiogenesis^15^. Further examination of factors known to be associated with angiogenesis (Fig. 2d, above and Supplementary Fig. S2b) and osteochondrogenesis (Fig. 2d, below) revealed that these genes exhibited significantly upregulated expression in *Mkx*^*−/−*^ tendons (Fig. 2d). Taken together, these findings indicate that genes related to angiogenesis and osteochondrogenesis are upregulated in *Mkx*^−*/*−^ tendons.

### Cellular profiles and transcriptomic signatures of WT and *Mkx*^−*/*−^tendons at the single-cell level

**Fig. 3:**
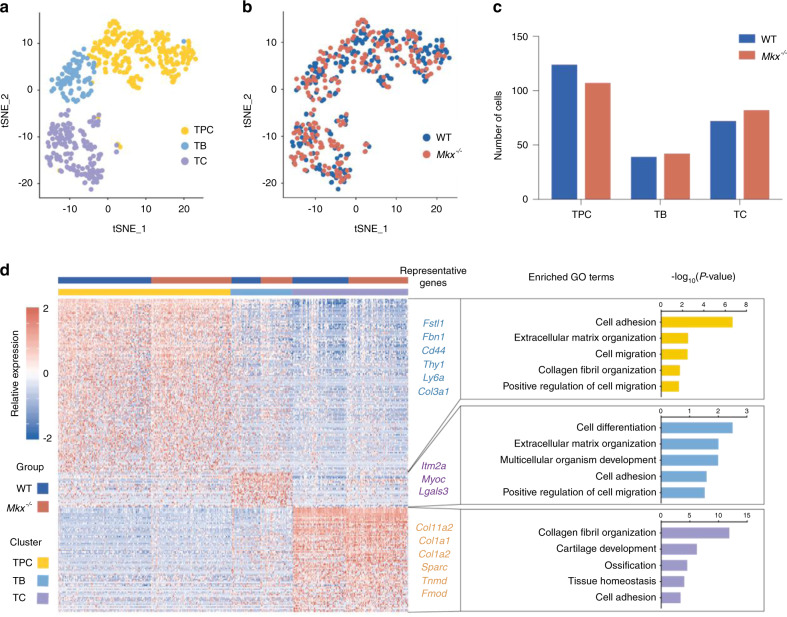
Single-cell profiles of WT and *Mkx*^*−/−*^ tendons. **a**, **b** t-SNE plot of WT and *Mkx*^*−/−*^ tendon cell clusters inferred from scRNA-seq data. The plots were visualized using CC1-CC4 and are colored by cell type (**a**) or genotype (**b**). CC: canonical vectors. **c** Numbers of WT and *Mkx*^*−/−*^ cells in each cell cluster. **d** Heatmap showing the marker genes for each cell cluster (left) and representative GO terms corresponding to these marker genes (right). TPCs tendon progenitor cells, TBs tenoblasts, TCs tenocytes

Given that tendon tissues are composed of different cell populations and that bulk RNA-seq revealed only the average expression levels in all cells, we next examined the expression profiles at the single-cell level to determine the cell populations contributing to the progression of HO. To do this, we isolated tail tendons from 4-week-old WT and *Mkx*^*−/−*^ mice and performed single-cell RNA-seq (scRNA-seq) using the Fluidigm C1 system with high-throughput integrated fluidic circuits (HT IFCs). In total, we captured 486 single cells, of which 466 cells passed our quality control criteria (materials and methods) and were included in further analyses. To compare WT and *Mkx*^*−/−*^ cells directly, we utilized a recently developed computational method (Seurat alignment workflow; SAW) for the integrated analysis of scRNA-seq data obtained under different conditions^16^. Unsupervised graph-based clustering was performed on the integrated dataset of both WT and *Mkx*^*−/−*^ cells, and the result was visualized using (t-distributed stochastic neighbor embedding (t-SNE)). Three distinct cell clusters were identified in both WT and *Mkx*^*−/−*^ tendons (Fig. 3a). More importantly, WT and *Mkx*^*−/−*^ cells were intermingled with each other in all three populations (Fig. 3b), suggesting that no population shift occurred in HO tendons at this stage. Consistently, the cell population compositions were comparable between WT and *Mkx*^*−/−*^ tendons (Fig. 3c). Taken together, the above analyses ruled out the possibility of cell population shifting as the main mechanism underlying HO progression.

To annotate the cells, marker genes representing each cluster were identified using Seruat^16^. For each cell cluster, we identified 126, 25, and 76 marker genes (Fig. 3d). Cells highly expressing markers of mesenchymal progenitor cells, such as *Cd44*, *Thy1*, and *Ly6a*, were annotated as tendon progenitor cells (TPCs) (Fig. 3d). TPCs also expressed high levels of *Fstl1* (Supplementary Fig. S3a), which has been reported to be upregulated at the onset of tenogenic mesenchymal stem cell differentiation^17^. Cells with high levels of *Itm2a*, a type II integral membrane protein involved in mesenchymal stem cell differentiation^18–20^, were annotated as tenoblasts (TBs) (Fig. 3d and Supplementary Fig. S3b). Cells enriched for extracellular matrix genes secreted by mature tendon cells, such as *Col11a2, Col1a1*, *Col1a2*, *Sparc, Tnmd and Fmod* (Fig. 3d and Supplementary Fig. S3c), were designated as tenocytes (TCs)^21–23^. We also validated the expression of one representative marker for each cell type (*Fstl1*, *Imt2a*, *Col1*) by immunofluorescence, confirming the existence of these cell types in both WT and *Mkx*^−*/*−^ tendons (Supplementary Fig. S3d). We then performed GO analyses of these marker genes in each cell type (Fig. 3d). TPCs were enriched for GO terms such as cell adhesion, extracellular matrix organization and cell migration, and TBs were enriched for the GO terms cell differentiation and cell adhesion. Intriguingly, TCs were enriched for GO terms such as collagen fibril organization, cartilage development and ossification, indicating that a proportion of tendon cells might easily initiate and contribute to endochondral bone formation (Fig. 3d). Overall, we identified three distinct cell subpopulations in both WT and *Mkx*^*−/−*^ tendons.

### Trajectory analyses reveal ectopic activation of osteochondrogenic genes in *Mkx*^*−/*−^ cells during tenogenesis

**Fig. 4:**
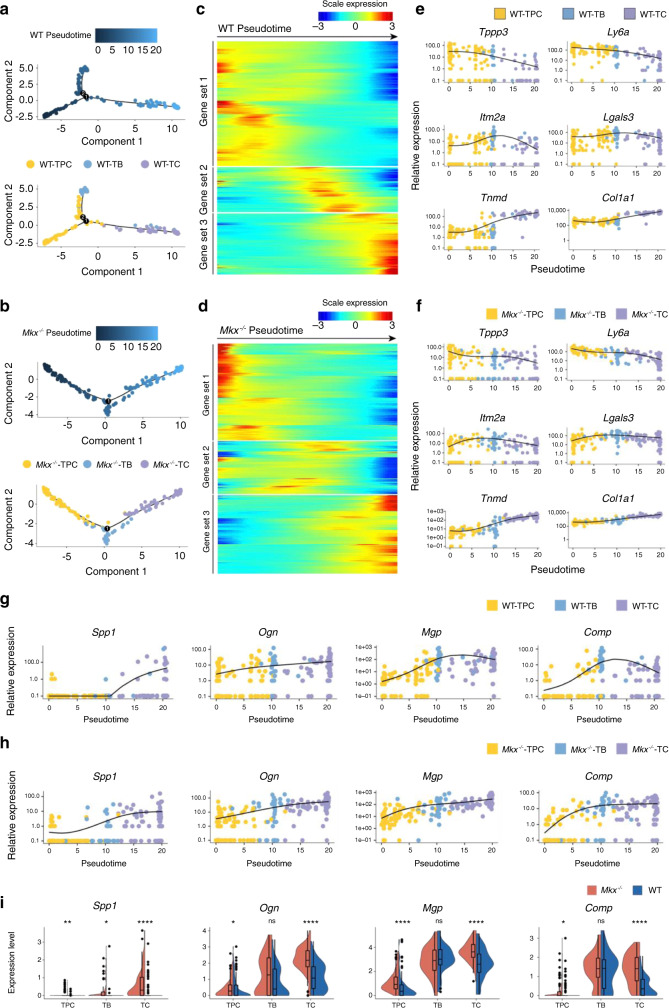
Pseudotime analysis revealed shifted gene expression programs in *Mkx*^*−/−*^ tendon cells. **a**, **b** Order of the WT (**a**) and *Mkx*^*−/−*^ (**b**) cells along the differentiation trajectory based on pseudotime values and cell clusters. **c**, **d** Gene expression dynamics along the WT (**c**) and *Mkx*^−*/−*^ (**d**) differentiation trajectories. Genes (row) are clustered and cells (column) are ordered according to pseudotime development. **e**, **f** Kinetic diagrams showing the expression of early, middle and late tenogenesis markers during the differentiation of WT (**e**) and *Mkx*^*−/−*^ (**f**) cells. **g**, **h** Kinetic diagrams showing the expression of osteochondrogenic markers during the differentiation of WT (**g**) and *Mkx*^*−/−*^ (**h**) cells. **i** Violin plots showing the *Spp1, Ogn*, *Mgp*, and *Comp* expression in wild-type and *Mkx*^*−/−*^ TPCs, TBs, and TCs. ns no significance; *P < 0.05; **P < 0.01; ****P < 0.000 1. TPCs tendon progenitor cells, TBs tenoblasts, TCs tenocytes

We next evaluated the gene expression dynamics during the heterotopic ossification process by analyzing the pseudotemporal trajectory using Monocle^24^. In both WT and *Mkx*^−*/*−^ tendon cells, we observed an enrichment of TPCs at the start of the pseudotemporal trajectory, which passed through an intermediate state dominated by TBs and ultimately transitioned to the terminal state, mainly consisting of TCs (Fig. 4a, b). Differentially expressed genes throughout WT or *Mkx*^*−/−*^ tendon differentiation were clustered into three gene sets, reflecting the early, middle and late differentiation stages (Fig. 4c, d). The pseudotemporal kinetics of representative early (*Ly6a*, *Tppp3*), middle (*Itm2a*, *Lgals3*) and late (*Tnmd*, *Col1a1*) stage markers showed similar expression patterns in WT and *Mkx*^*−/−*^ cells, suggesting that the key gene expression program required for tenogenesis was, to a certain extent, maintained in *Mkx*^*−/−*^ cells (Fig. 4e, f).

We next examined whether the expression levels of osteochondrogenic genes were altered in *Mkx*^*−/−*^ tendon cells. Interestingly, osteochondrogenic marker genes (*Spp1*, *Ogn*, *Mgp*, and *Comp*) showed similar increasing expression trends from the early to the late stage of differentiation in WT and *Mkx*^*−/−*^ tendon cells (Fig. 4g, h). Nevertheless, the upregulation of osteogenic genes seemed to occur earlier in *Mkx*^*−/−*^ cells than in WT cells (Fig. 4g, h). In addition, the expression levels of these genes were relatively higher in *Mkx*^*−/−*^ cell clusters than in WT cells (Fig. 4i). To further investigate the changes in the osteochondrogenic gene expression program in *Mkx*^*−/−*^ cells, we performed gene set score analysis to determine whether a specific set of genes was expressed in a specific cell type at higher than expected levels. We found that the expression levels of genes associated with osteoblast development were significantly increased in *Mkx*^*−/−*^ TPCs, while genes linked to ossification and bone mineralization were highly expressed in both WT and *Mkx*^*−/−*^ TBs and TCs (Supplementary Fig. S4a, b and Supplementary Table S1). Collectively, these analyses indicated that the heterotopic ossification of *Mkx*^*−/−*^ tendons was associated with the excessive activation of osteochondrogenic genes as early as the TPC stage and not with the de novo activation of these genes.

### Increased angiogenic gene expression and vascularity in *Mkx*^*−/−*^ tendons

**Fig. 5:**
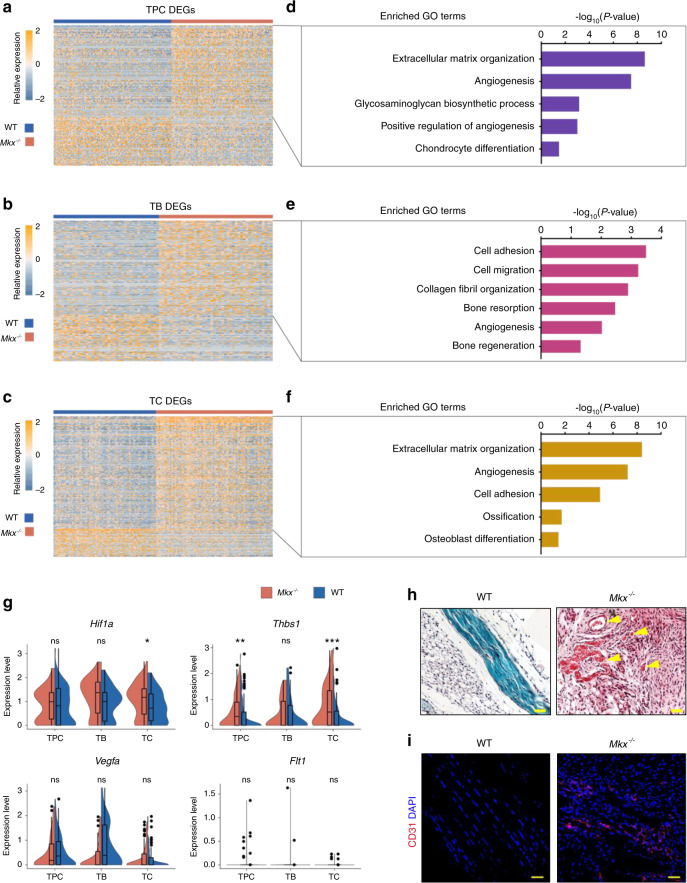
Differential gene expression analysis revealed elevated angiogenic gene expression in *Mkx*^*−/−*^ tendon cells. **a**–**c** Heatmap of the normalized gene expression of DEGs between WT and *Mkx*^*−/−*^ TPC (**a**), TB (**b**) and TC (**c**) clusters. **d**–**f** Representative GO terms corresponding to upregulated genes in the *Mkx*^*−/−*^ TPC (**d**), TB (**e**) and TC (**f**) clusters. **g** Violin plots of proangiogenic genes, including *Hif1a, Thbs1, Vegfa*, and *Flt1*, in the TPC, TB and TC clusters. **h** Safranin O-Fast green staining of the patellar tendons from WT and *Mkx*^*−/−*^ mice. The yellow arrows indicate the blood vessels. Scale bar, 50 μm. **i** Immunostaining of CD31 in WT and *Mkx*^*−/−*^ patellar tendons. Scale bar, 50 μm. ns no significance; *P < 0.05; **P < 0.01; ***P < 0.001. TPCs tendon progenitor cells, TBs tenoblasts, TCs tenocytes

To further explore the effects of *Mkx* knockout on the gene expression levels in different cell types, we analyzed the DEGs between WT and *Mkx*^*−/−*^ tendons for each cell cluster (Fig. 5a–c). We performed GO term analysis of genes that were upregulated in *Mkx*^−*/−*^ cells for each cell type (Fig. 5d–f). Interestingly, angiogenesis-related genes were consistently enriched in all three cell types of *Mkx*^*−/−*^ tendons (Fig. 5d–f), which was confirmed by our observations at the bulk level (Fig. 2b, d). Specifically, violin plots showed that *Hif1a* was highly expressed in *Mkx*^*−/−*^ TCs, and *Thbs1* was highly expressed in *Mkx*^*−/−*^ TPCs and TCs (Fig. 5g). The expression of *Vegfa* and *Flt1* was comparable among each cell cluster, indicating that Vegfa/Vegfr signaling was not activated at this stage (Fig. 5g). SOFG and immunofluorescence staining of CD31 also showed increased blood vessels in *Mkx*^*−/−*^ tendons (Fig. 5h, i). Altogether, these findings indicate that the progression of tendon HO is highly coupled with the upregulation of angiogenesis-related genes and increased vascularity.

### The angiogenesis inhibitor BIBF1120 attenuates the progression of *Mkx*^*−/*^^−^ and trauma-induced HO

**Fig. 6:**
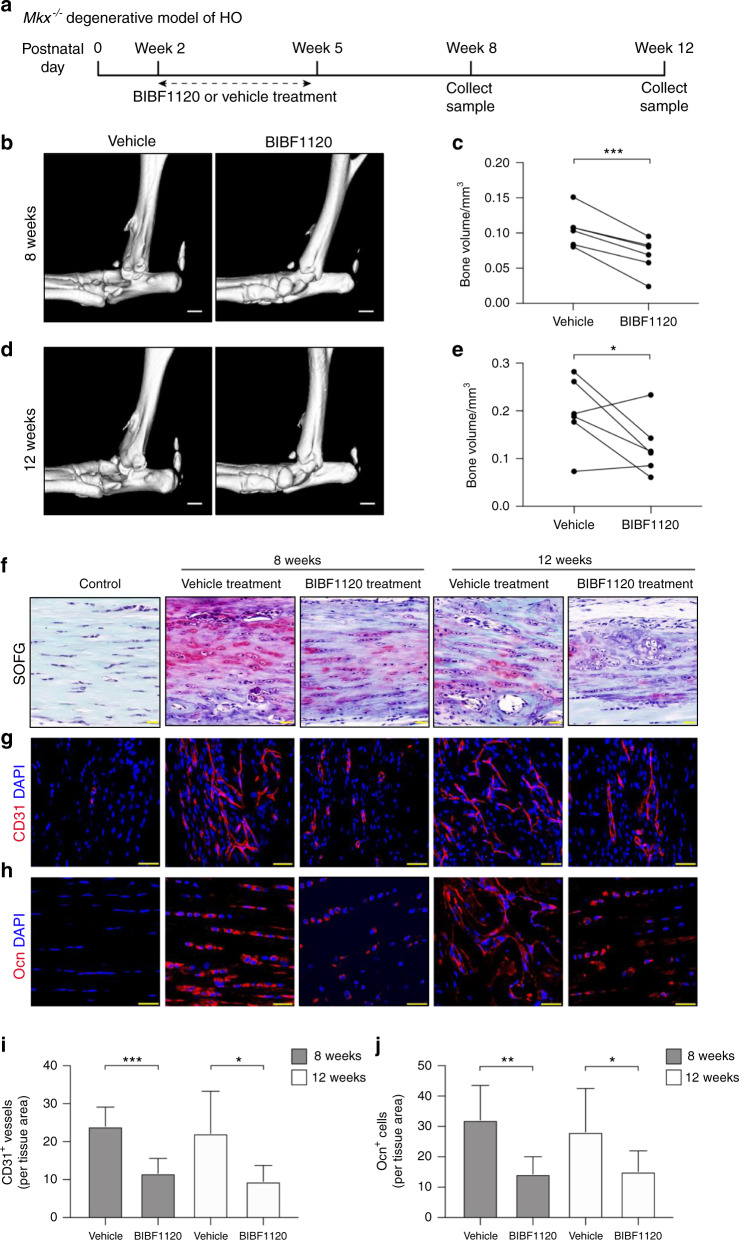
The angiogenesis inhibitor BIBF1120 attenuates heterotopic bone formation in the *Mkx*^*−/−*^ degenerative model of HO. **a** Schematic illustration of the treatment and sample collection workflow. **b**–**e** Micro-CT images of the Achilles tendons of 8-week-old (**b**) and 12-week-old (**d**) *Mkx*^*−/−*^ mice treated with BIBF1120 or vehicle. Scale bar, 1 mm. Quantitative analysis of the heterotopic bone volumes of the Achilles tendons of 8-week-old (**c**) and 12-week-old (**e**) *Mkx*^*−/−*^ mice. **f** Safranin O-Fast green staining of the Achilles tendons of normal, 8-week-old and 12-week-old *Mkx*^*−*^^*/*−^ mice. Scale bar, 50 μm. **g** Immunostaining of CD31^+^ (red) vessels. Scale bar, 50 μm. **h** Immunostaining of Ocn^+^ (red) cells in ectopic bone marrow. Scale bar, 25 μm. **i**, **j** Quantification of CD31^+^ (**i**) vessels and Ocn^+^ (**j**) cells. All data are shown as the mean ± s.d. *n* = 6 per group. Statistical test: student *t* test. *P < 0.05; **P < 0.01; ***P < 0.001

We next investigated whether the inhibition of angiogenesis would attenuate HO progression. The angiogenesis inhibitor BIBF1120 or vehicle (solvent used to dissolve BIBF1120, as a negative control) was injected into the Achilles tendons of *Mkx* knockout mice three times a week for three consecutive weeks from postnatal day 14. The mice were sacrificed at the age of 8 or 12 weeks for further characterization (Fig. 6a). The micro-CT results showed that bone formation was significantly mitigated by BIBF1120 treatment at both time points (Fig. 6b–e). SOFG staining revealed larger and well-developed cancellous bones and marrow in the vehicle group (Fig. 6f). Significantly more CD31^+^ vessels were observed in the vehicle group than in the BIBF1120 group (Fig. 6g, i). In addition, the Ocn^+^ osteoblast number was significantly lower in the BIBF1120 group than in the vehicle group (Fig. 6h, j). Altogether, these results indicated that the inhibition of angiogenesis by BIBF1120 alleviated HO progression in *Mkx*^*−/−*^ tendons.

**Fig. 7:**
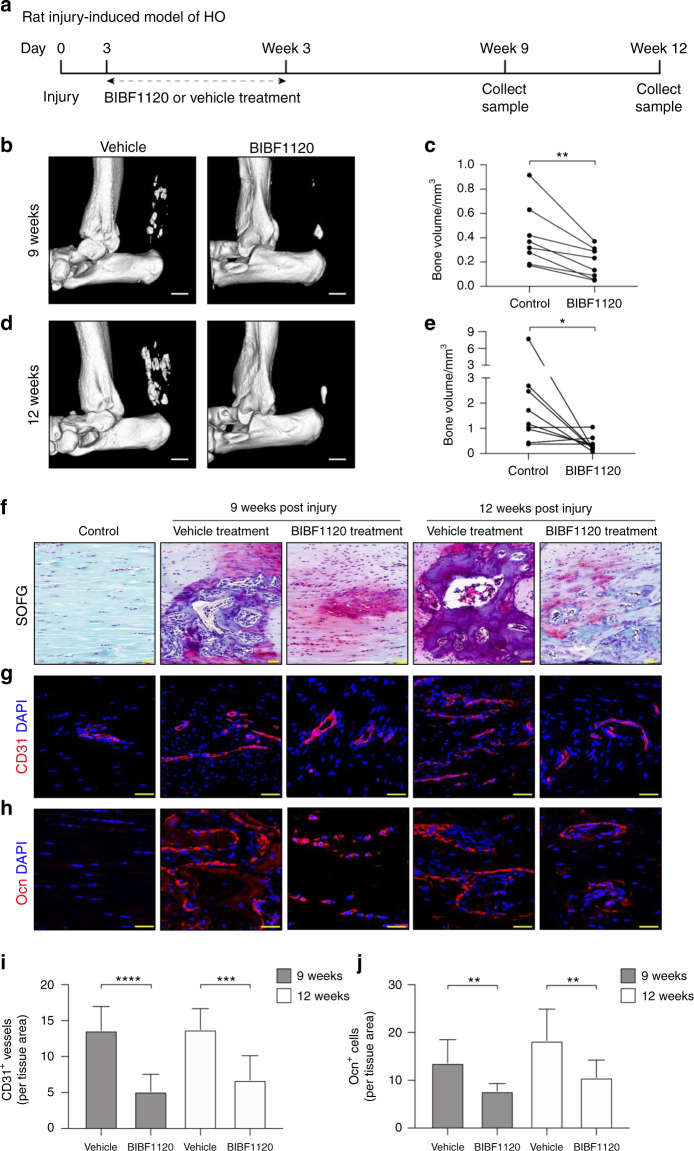
The angiogenesis inhibitor BIBF1120 attenuates heterotopic bone formation in a trauma-induced model of rat HO. **a** Schematic illustration of the treatment and sample collection workflow for the rat HO model. **b**–**e** Micro-CT images of the Achilles tendons of rats in the BIBF1120 and vehicle treatment groups at 9 (**b**) and 12 weeks (**d**) post ATP. Scale bar, 1 mm. Quantitative analysis of the heterotopic bone volumes of the Achilles tendons at 9 (**c**) and 12 weeks (**e**) postinjury. **f** Safranin O-Fast green staining of the rat Achilles tendons of normal rats at 9 and 12 weeks postinjury. Scale bar, 50 μm. **g** Immunostaining of CD31^+^ (red) vessels. Scale bar, 50 μm. **h** Immunostaining of Ocn^+^ (red) cells in ectopic bone marrow. Scale bar, 25 μm. **i**, **j** Quantification of CD31^+^ (**i**) vessels and Ocn^+^ (**j**) cells. All data are shown as the mean ± s.d. *n* = 8 per group. Statistic test: student *t* test. **P < 0.01; ***P < 0.001; ****P < 0.000 1

However, the *Mkx*^*−/*−^ HO model represents a degenerative model in which the animals were not subjected to any trauma. However, in the clinic, tendon HO is often caused by trauma^25^. To better simulate clinical tendon HO, we constructed a trauma-induced HO model in rats based on percutaneous Achilles tendon puncture (ATP)^3^. We found that the expression of Mkx was also downregulated in the trauma-induced HO model (Supplementary Fig. S5). We then treated the trauma-induced HO model rats with BIBF1120 or vehicle. Each rat received a subcutaneous injection (between the Achilles tendon and skin) of BIBF1120 or vehicle three times a week for three weeks from the third day after injury and was sacrificed at 9 or 12 weeks postinjury (Fig. 7a). Micro-CT and SOFG staining analyses showed that the HO formation and bone volume were significantly reduced in the BIBF1120-treated group compared with the control group at 9 and 12 weeks after ATP (Fig. 7b–f). Importantly, the immunofluorescence staining results showed that CD31^+^ vessels were reduced in the BIBF1120 group relative to the vehicle control group (Fig. 7g, i). In addition, the number of Ocn^+^ osteoblasts was significantly reduced upon BIBF1120 treatment (Fig. 7h, j). Collectively, these results demonstrated that the inhibition of angiogenesis with the small molecule BIBF1120 attenuated HO progression.

However, BIBF1120 targets various signaling pathways, including the Vegfr, Pdgfr and Fgfr signaling pathways, which are also important for the maintenance of tendon homeostasis^26–28^. To determine whether BIBF1120 treatment affects tendon cell proliferation and differentiation, we performed a bromodeoxyuridine (BrdU) assay and RNA-seq analysis of rat tendons treated with BIBF1120 and vehicle. Immunofluorescence staining showed similar levels of BrdU-positive cells in the BIBF1120- and vehicle-treated groups (Supplementary Fig. S6a, b). Consistently, the examination of proliferation marker genes (*Mki67*, *Pcna*, *Mcm2*) showed that their expression levels were comparable between the BIBF1120 and vehicle groups (Supplementary Fig. S6d). In addition, the gene expression levels at the genome-wide scale were highly correlated between the BIBF1120- and vehicle-treated groups, suggesting that BIBF1120 minimally effects the transcript profiles of tendons (Supplementary Fig. S6c). Importantly, we also observed similar expression levels of tendon stem/progenitor cell markers (*Mcam*, *Thy1*, *Nes*) and other canonical tendon markers (*Scx*, *Mkx*, *Egr1*, *Tnmd*, *Tnc* and *Col1a1*) between the BIBF1120 and vehicle groups, indicating that tendon homeostasis and differentiation were not obviously affected by BIBF1120 (Supplementary Fig. S6e).

## Discussion

The limited understanding of tendon HO and the urgent need for an effective therapeutic motivated us to investigate its pathogenesis and to develop potential novel therapeutic strategies. Here, we used *Mkx*^−*/−*^ mice as the tendon HO model to study the underlying mechanisms. Our data revealed a previously unknown role of Mkx in regulating the fates of different cell subpopulations. These high-throughput sequencing data suggested the inhibition of angiogenesis as a potential therapeutic target for attenuating tendon HO, which inspired us to assess the effect of the small-molecule angiogenesis inhibitor BIBF1120 on the progression of tendon HO. Our data demonstrated that treatment with BIBF1120 displayed promising effects on attenuating the progression of degenerative and trauma-induced tendon HO.

Recently, an increasing number of studies have used single-cell analysis to explore the roles of different cell subpopulations in tendon development and pathology^7,8,29^. Using the highly parallel strategy of single-cell quantitative reverse transcription polymerase chain reaction (single-cell qRT–PCR), we previously identified three subpopulations of human tendon stem/progenitor cells (TSPCs) after in vitro expansion and confirmed that the Nestin-positive subpopulation possesses the best tendon regeneration capability^7^. Our single-cell analyses presented herein did not recapitulate the cell types we previously identified due to the species differences and to the potential selective effects of in vitro culture on the cell subpopulations. Although our data indicated that TPCs may gain osteogenic differentiation potential upon *Mkx* knockout, further studies are needed to trace their precise fate. Moreover, other cell populations, such as endothelial cells, smooth muscle cells and pericytes, which are important for angiogenesis, were not identified in our single-cell analysis, probably due to the limited number of cells captured. Further analyses using a high-throughput single-cell method may provide a more comprehensive landscape of the transcriptional heterogeneity of tendon cells under normal and heterotopic ossification conditions.

Angiogenesis and osteogenesis processes are highly coupled and are both critical for skeletal development^30^. However, neovascularization may have adverse effects on tissue repair. A recent study showed that the excessive activation of TGF-β signaling promoted osteogenesis and neovascularization during HO progression^3^. Cocks and colleagues analyzed 29 human HO specimens and found that human HO exhibited a time- and space-dependent pattern of vascularization^31^. Our results showed that angiogenesis-related genes were upregulated in an *Mkx*^*−/−*^ HO mouse model, and neovascularization was validated by histological analyses. Thus, we hypothesized that inhibiting neovascularization may attenuate tendon heterotopic ossification. In this study, we found that the small-molecule drug BIBF1120 significantly inhibited bone formation and vascularity in both *Mkx*^*−/−*^ mice and a trauma-induced model of HO. BIBF1120 is a triple vascular kinase inhibitor that can effectively suppress the proangiogenic signaling pathway mediated by Vegfr, Pdgfr and Fgfr in vascular endothelial cells, perivascular skin cells and smooth muscle cells^26^. In addition to mediating the proangiogenic signaling pathway, vegfr, pdgfr, and fgfr are involved in signaling pathways that regulate many aspects of cell biology, such as cell proliferation, migration and fate determination^32–34^. In tendon biology, for example, Fgf/Fgfr was the first signaling pathway identified to induce tendon cells in vivo, and Pdgf/Pdgfr signaling plays an important role in postnatal tendon growth^27,28^. Thus, the paninhibition of these signaling pathways by BIBF1120 could have adverse effects on surrounding tendon cells that are not specific to vascular blockade. Nevertheless, our results showed that the treatment of tendons with BIBF1120 had minimal side effects on the proliferation and expression of key marker genes, showing a relatively specific function of BIBF1120 in preventing tendon HO and highlighting its promising application potential.

In summary, this study revealed the mechanism underlying tendon HO by using an *Mkx* knockout model and potential provides a clinically applicable therapeutic approach to attenuate tendon HO through the inhibition of angiogenesis with BIBF1120. These findings will be of great value for elucidating the pathogenesis of tendon HO as well as for the development of novel therapeutic approaches for heterotopic ossification induced by tendon injury and surgery.

## Materials and methods

### Animals

*Mkx*^*−/−*^ transgenic mice were provided by Dr. Ronen Schweitzer (Oregon Health and Science University, Portland, OR). All animal procedures in this study were approved by the Institutional Animal Care and Use Committee of Zhejiang University.

### Histological and histochemical examination

For histological analysis, samples were fixed in 4% (v/v) paraformaldehyde for 24 hours and then decalcified in 10% (w/v) EDTA-decalcification solution for 20 days. Achilles tendons were fixed in 4% (v/v) paraformaldehyde for 24 hours. Then, the samples were dehydrated through an alcohol gradient, cleared, and embedded in paraffin blocks. Histological sections (6 μm) were prepared using a microtome and subsequently processed for safranin O-fast green staining and immunostaining. Immunohistochemical analysis was performed in accordance with the standard protocol. Sections were incubated overnight at 4 °C with primary antibodies and then incubated at room temperature for 2 h with secondary antibodies. For immunofluorescence, the following antibodies were used: anti-MKX (1:100, LifeSpan, LS-B8063), anti-OCN (1:100), anti-FSTL1 (1:100, Proteintech, 20182-1-AP), anti-ITM2A (1:100, Proteintech, 18306-1-AP), anti-COL1 (1:100, Abcam, ab34710), anti-CD31 (1:100, Abcam, ab222783), anti-BrdU (1:150, Proteintech, 66241-1-Ig), and secondary antibodies conjugated with Alexa Fluor 488 and 546 (1:500, Thermo Fisher Scientific). The stained specimens were photographed digitally under a fluorescence microscope.

### Microcomputed tomography imaging and analyses

Hind limbs and knee joints were dissected from mice and rats, fixed overnight in 4% paraformaldehyde and analyzed by MILabs U-SPECT4CT with default parameters.

### Bulk RNA-seq and data analysis

For the bulk population, total RNA was extracted from tail tendons using TRIzol reagent (Invitrogen, Carlsbad, CA, USA), and a cDNA library was prepared using the Ion Total RNA-Seq Kit v2 (Life Technologies Corporation, Carlsbad, USA) according to the manufacturer’s protocol. RNA sequencing was performed on the Ion Proton system. Clean reads were obtained from the raw reads by removing the adaptor sequences, reads with >5% ambiguous bases (noted as N) and low-quality reads containing more than 20% of bases with a quality score <13. The clean reads were then aligned to the mouse genome (mm9) using the MapSplice program (v2.1.6). We applied the DESeq2 algorithm to filter the differentially expressed genes with the following criteria: (i) log2(fold change) > 1 or < −1 and (ii) padj < 0.05. Gene ontology (GO) analysis was performed using DAVID^35,36^.

### Single-cell capture, cDNA library preparation and sequencing

First, tail tendons from 4-week-old WT or *Mkx*^−*/−*^ mice were minced into desired pieces and digested with 0.2% collagenase I (Life Technologies) diluted in low-glucose Dulbecco’s modified Eagle’s medium (Gibco) at 37 °C for 2 h. The resulting cell suspension was filtered using a 40 μm strainer to remove the incompletely digested clumps and adjusted to a concentration of 4 × 10^5^ cells per mL. Single-cell capture, RNA extraction and cDNA preparation were performed in accordance with methods described in the Fluidigm protocol (PN 100-9886, Using C1 High-Throughput IFC to Generate Single-cell cDNA Libraries for mRNA Sequencing). The cDNA reaction products were quantified using a Qubit fluorometer and were then diluted to a final concentration of 0.2 ng·μL^−1^ using C1 Harvest Reagent. The diluted cDNA reaction products were then converted into mRNA-seq libraries using the Nextera XT DNA Sample Preparation Kit (Illumina, FC-131-1096, -2001 and -2002) in accordance with the manufacturer’s instructions. The library was sequenced by Annoroad (Beijing, China) on an Illumina HiSeq X Ten platform (Illumina, Santiago, CA, USA), and 150 bp paired-end reads were generated.

### Single cell RNA-seq data analysis

Single-cell RNA sequencing reads were evaluated by AfterQC^37^. Bad reads were detected automatically and removed. Bases at the 3ʹ end of the read were further trimmed by NGSQCToolkit if their PHRED quality score was less than 20^38^. The reads were mapped to the mm9 genome using bowtie2. The mapped reads for genes were counted with featureCounts^39^. Quality control, clustering and differential expression analysis were performed using Seurat v2.3.0 in accordance with a recently published method^16,40^. For integrated analysis, the union set of the top 1 000 highly variable genes in WT and *Mkx*^*−/−*^ cells was used to perform a canonical correlation analysis, and 20 cells that were not well described by a shared gene correlation structure were excluded. Four principal components were used for the alignment of the CCA subspaces and for cell clustering at a resolution of 0.4. The marker genes of each cell cluster were analyzed using the FindConservedMarkers function with the *test.use* = *“wilcox”* option and the logfc.threshold parameter set to 0.5. Cell type-specific differentially expressed genes were identified using the FindMarkers function with default parameters. Pseudotime analysis was performed with Monocle v2.6.3 using digital gene expression matrices with annotations from Seurat as the input^24^.

### Gene set enrichment analysis

The module scores for gene expression programs in single cells were calculated using the *AddModuleScore* function of the Seurat package^41^. First, all the analyzed genes were binned based on the average expression, and the control genes were randomly selected from each bin. Then, the average expression value of the gene set at the single-cell level minus the aggregated expression of the control gene set was calculated. Gene sets were obtained from the Mgi database (http://www.informatics.jax.org/mgihome/GO).

### Animal experiments

For the *Mkx*^*−/−*^ mouse HO model, each 2-week-old mouse was subcutaneously injected (between the Achilles tendon and skin) with 8 µL of BIBF1120 (4 μg) in the right leg and 8 µL of vehicle (an identical solvent used to dissolve BIBF1120) in the left leg three times a week for three weeks. The mice were euthanized at 8 or 12 weeks old for micro-CT imaging and histological analysis.

For the rat traumatic model of HO, eight-week-old male rats were anesthetized by isoflurane. A 27-gauge needle was used to puncture the Achilles tendon body from the lateral aspect percutaneously, and this process was repeated twenty-five times at five different parts of the Achilles tendon body for each rat. Three days postinjury, each rat was subcutaneously injected with 40 µL of BIBF1120 (40 μg) in the right leg and 40 µL of vehicle in the left leg three times a week for three weeks. At 9 or 12 weeks after treatment, the rats were euthanized for micro-CT imaging and histological analysis.

Cell proliferation in vivo was detected as previously described^42^. Specifically, BrdU was reconstituted in DMSO as a stock (80 mmol·L^−1^), and 150 μL of BrdU mixed with 350 μL of PBS was injected intraperitoneally into the rats together with BIBF1120 and the vehicle. Tissue sections were treated with 1.5 mol·L^-1^ HCl at room temperature for 0.5 h, and DNA synthesis was assessed with an anti-BrdU antibody.

### Statistical analysis

All statistical analyses were performed with GraphPad Prism software. The means and standard deviations were calculated from numerical data and are presented in the text and figures. Significant differences in the mean values were determined using Student’s *t* test unless indicated otherwise. *P* < 0.05 was considered significant. Statistical significance is indicated by *P < 0.05, **P < 0.01, ***P < 0.001, and ****P < 0.000 1.

## Supplementary information

Supplementary Table 1

Supplementary Data

## Data Availability

The single-cell and bulk RNA-seq data utilized in this study are deposited into the Gene Expression Omnibus (GEO) under accession number GSE102931.

## References

[1] Shehab D, Elgazzar AH, Collier BD (2002). Heterotopic ossification. J. Nucl. Med.

[2] Meyers C (2019). Heterotopic ossification: a comprehensive review. JBMR Plus.

[3] Wang X (2018). Inhibition of overactive TGF-beta attenuates progression of heterotopic ossification in mice. Nat. Commun..

[4] Agarwal S (2017). Scleraxis-lineage cells contribute to ectopic bone formation in muscle and tendon. Stem Cells.

[5] Bi Y (2007). Identification of tendon stem/progenitor cells and the role of the extracellular matrix in their niche. Nat. Med..

[6] Lee CH (2015). Harnessing endogenous stem/progenitor cells for tendon regeneration. J. Clin. Invest.

[7] Yin Z (2016). Single-cell analysis reveals a nestin+ tendon stem/progenitor cell population with strong tenogenic potentiality. Sci. Adv..

[8] Harvey T, Flamenco S, Fan CM (2019). A Tapp3^+^ Pdgfra^+^ tendon stem cell population contributes to regeneration and reveals a shared role for PDGF signalling in regeneration and fibrosis. Nat. Cell Biol..

[9] Ito Y (2010). The Mohawk homeobox gene is a critical regulator of tendon differentiation. Proc. Natl. Acad. Sci. USA.

[10] Kimura W (2011). Irxl1 mutant mice show reduced tendon differentiation and no patterning defects in musculoskeletal system development. Genesis.

[11] Liu H (2015). Mohawk promotes the tenogenesis of mesenchymal stem cells through activation of the TGFbeta signaling pathway. Stem Cells.

[12] Liu W (2010). The atypical homeodomain transcription factor Mohawk controls tendon morphogenesis. Mol. Cell Biol..

[13] Suzuki H (2016). Gene targeting of the transcription factor Mohawk in rats causes heterotopic ossification of Achilles tendon via failed tenogenesis. Proc. Natl. Acad. Sci. USA.

[14] Liu H, Xu J, Jiang R (2019). Mkx-deficient mice exhibit hedgehog signaling-dependent ectopic ossification in the achilles tendons. J. Bone Min. Res..

[15] Dejana E (2010). The role of wnt signaling in physiological and pathological angiogenesis. Circ. Res.

[16] Butler, A., Hoffman, P., Smibert, P., Papalexi, E. & Satija, R. Integrating single-cell transcriptomic data across different conditions, technologies, and species. *Nat. Biotechnol*. **36**, 411–420 (2018). 10.1038/nbt.4096 PMC6700744 29608179

[17] Noack S (2014). Periostin secreted by mesenchymal stem cells supports tendon formation in an ectopic mouse model. Stem Cells Dev..

[18] Van den Plas D, Merregaert J (2004). Constitutive overexpression of the integral membrane protein Itm2A enhances myogenic differentiation of C2C12 cells. Cell Biol. Int.

[19] Van den Plas D, Merregaert J (2004). In vitro studies on Itm2a reveal its involvement in early stages of the chondrogenic differentiation pathway. Biol. Cell.

[20] Boeuf S (2009). Enhanced ITM2A expression inhibits chondrogenic differentiation of mesenchymal stem cells. Differentiation.

[21] Wenstrup RJ (2011). Regulation of collagen fibril nucleation and initial fibril assembly involves coordinate interactions with collagens V and XI in developing tendon. J. Biol. Chem..

[22] Docheva D, Hunziker EB, Fassler R, Brandau O (2005). Tenomodulin is necessary for tenocyte proliferation and tendon maturation. Mol. Cell Biol..

[23] Gehwolf R (2016). Pleiotropic roles of the matricellular protein Sparc in tendon maturation and ageing. Sci. Rep..

[24] Qiu X (2017). Single-cell mRNA quantification and differential analysis with Census. Nat. Methods.

[25] Zhang Q, Zhou D, Wang H, Tan J (2020). Heterotopic ossification of tendon and ligament. J. Cell Mol. Med.

[26] Hilberg F (2008). BIBF 1120: triple angiokinase inhibitor with sustained receptor blockade and good antitumor efficacy. Cancer Res..

[27] Huang AH, Lu HH, Schweitzer R (2015). Molecular regulation of tendon cell fate during development. J. Orthop. Res.

[28] Sugg KB (2018). Postnatal tendon growth and remodeling require platelet-derived growth factor receptor signaling. Am. J. Physiol. Cell Physiol..

[29] Tan, G. K. et al. Tgfbeta signaling is critical for maintenance of the tendon cell fate. *Elife* **9**, e52695 (2020). 10.7554/eLife.52695 PMC7025861 31961320

[30] Kusumbe AP, Ramasamy SK, Adams RH (2014). Coupling of angiogenesis and osteogenesis by a specific vessel subtype in bone. Nature.

[31] Cocks M (2017). Vascular patterning in human heterotopic ossification. Hum. Pathol..

[32] Olsson AK, Dimberg A, Kreuger J, Claesson-Welsh L (2006). VEGF receptor signalling - in control of vascular function. Nat. Rev. Mol. Cell Biol..

[33] Andrae J, Gallini R, Betsholtz C (2008). Role of platelet-derived growth factors in physiology and medicine. Genes Dev..

[34] Ornitz DM, Itoh N (2015). The fibroblast growth factor signaling pathway. Wiley Interdiscip. Rev. Dev. Biol..

[35] Huang da W, Sherman BT, Lempicki RA (2009). Bioinformatics enrichment tools: paths toward the comprehensive functional analysis of large gene lists. Nucleic Acids Res..

[36] Huang da W, Sherman BT, Lempicki RA (2009). Systematic and integrative analysis of large gene lists using DAVID bioinformatics resources. Nat. Protoc..

[37] Chen S (2017). AfterQC: automatic filtering, trimming, error removing and quality control for fastq data. BMC Bioinforma..

[38] Patel RK, Jain M (2012). NGS QC Toolkit: a toolkit for quality control of next generation sequencing data. PLoS One.

[39] Liao Y, Smyth GK, Shi W (2014). featureCounts: an efficient general purpose program for assigning sequence reads to genomic features. Bioinformatics.

[40] Satija R, Farrell JA, Gennert D, Schier AF, Regev A (2015). Spatial reconstruction of single-cell gene expression data. Nat. Biotechnol..

[41] Zou Z (2021). A single-cell transcriptomic atlas of human skin aging. Dev. Cell.

[42] Ho TC (2019). PEDF-derived peptide promotes tendon regeneration through its mitogenic effect on tendon stem/progenitor cells. Stem Cell Res. Ther..

