# Supplementary material for: Single cell analysis reveals inhibition of angiogenesis attenuates the progression of heterotopic ossification in Mkx−/− mice
Source: Bone Res. 2022 Jan 7;10:4. doi: 10.1038/s41413-021-00175-9 (PMC8741758; doi:10.1038/s41413-021-00175-9)
Supplement: Supplementary file 2 — Supplementary Data [file 41413_2021_175_MOESM2_ESM.docx]

**Supplementary Data**

Supplementary Figures and Figure Legends

**Supplementary Fig. S1**


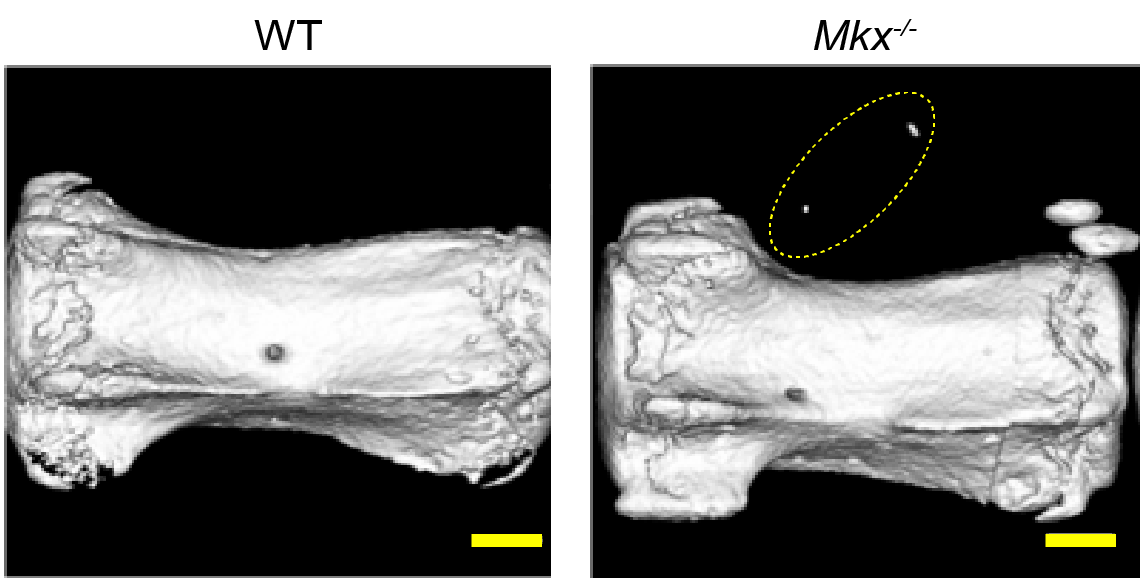


**Supplementary Fig. S1.** MicroCT analysis of WT and *Mkx^-/-^* tail tendons. MicroCT examination of tail tendons from WT and *Mkx^-/-^* mice. The dotted circle indicates the heterotopic bone. Scale bar, 1 mm.

**Supplementary Fig. S2**

**
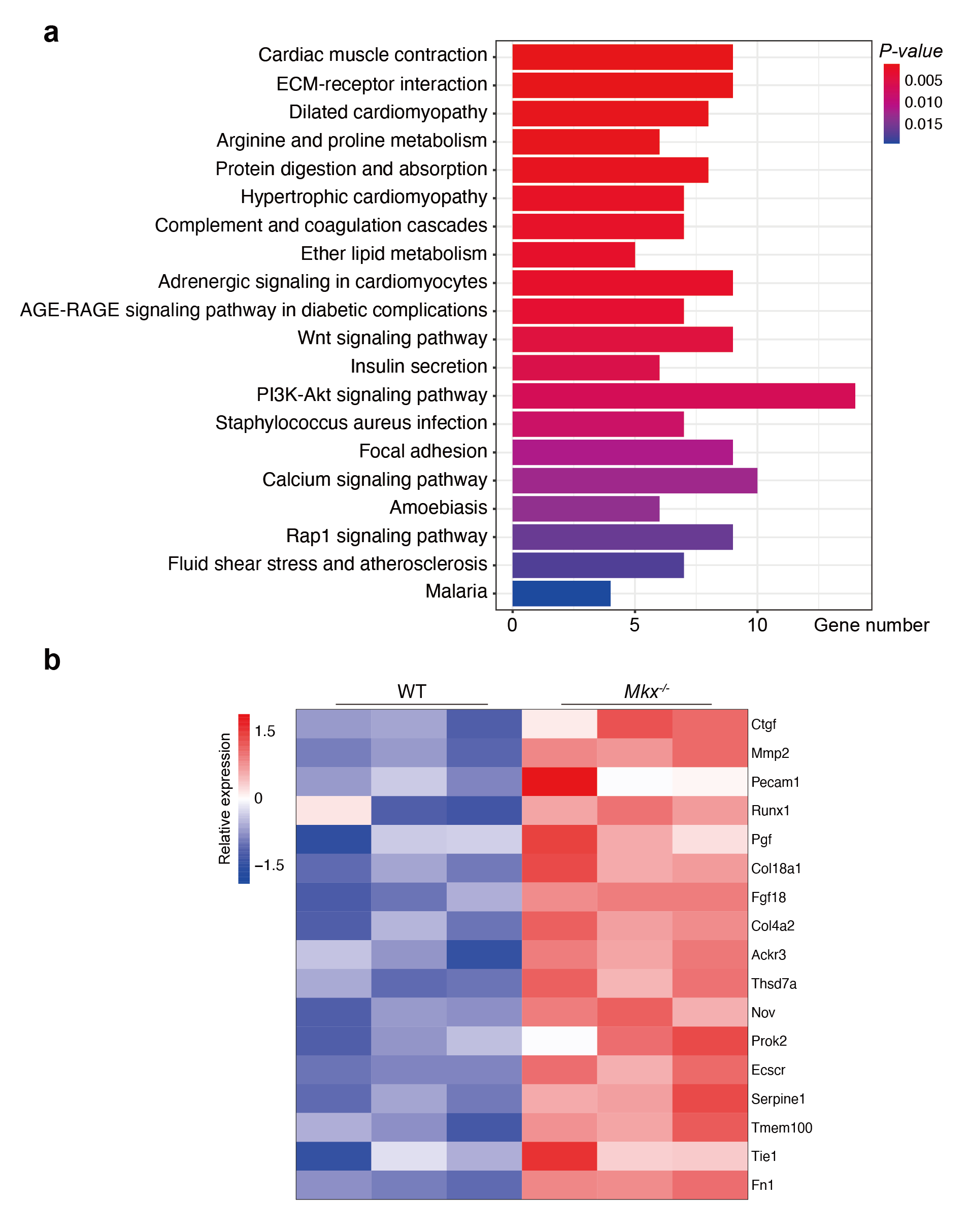
**

**Supplementary Fig. S2.** KEGG pathway analysis and heatmap plot of genes upregulated upon *Mkx* knockout. **a** Top 20 significantly enriched KEGG pathways. The color of the bar denotes the P value. X-axis: Number of genes related to the enriched KEGG terms. **b** Heatmap showing the expression of known angiogenic factors.

**Supplementary Fig. S3**


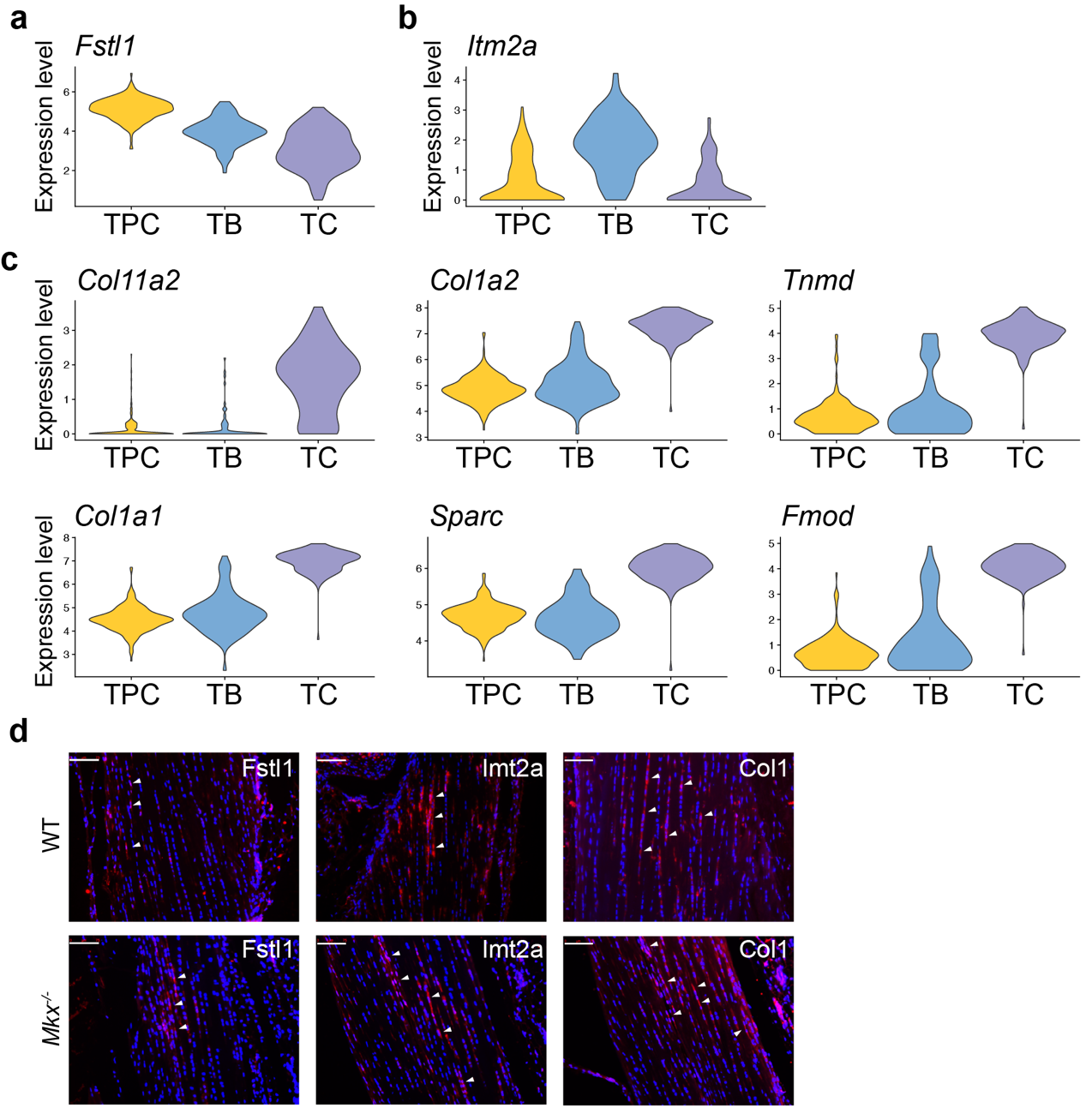


**Supplementary Fig. S3.** Violin plots and immunofluorescence staining of representative marker genes in each cell cluster. **a-c** Violin plot of the TPC marker Fstl1 (**a**), TB marker Itm2a (**b**) and TC markers (**c**). **d** Immunofluorescence staining of Fstl1, Itm2a and Col1 in Achilles tendons. Scale bar: 75 μm.

**Supplementary Fig. S4**


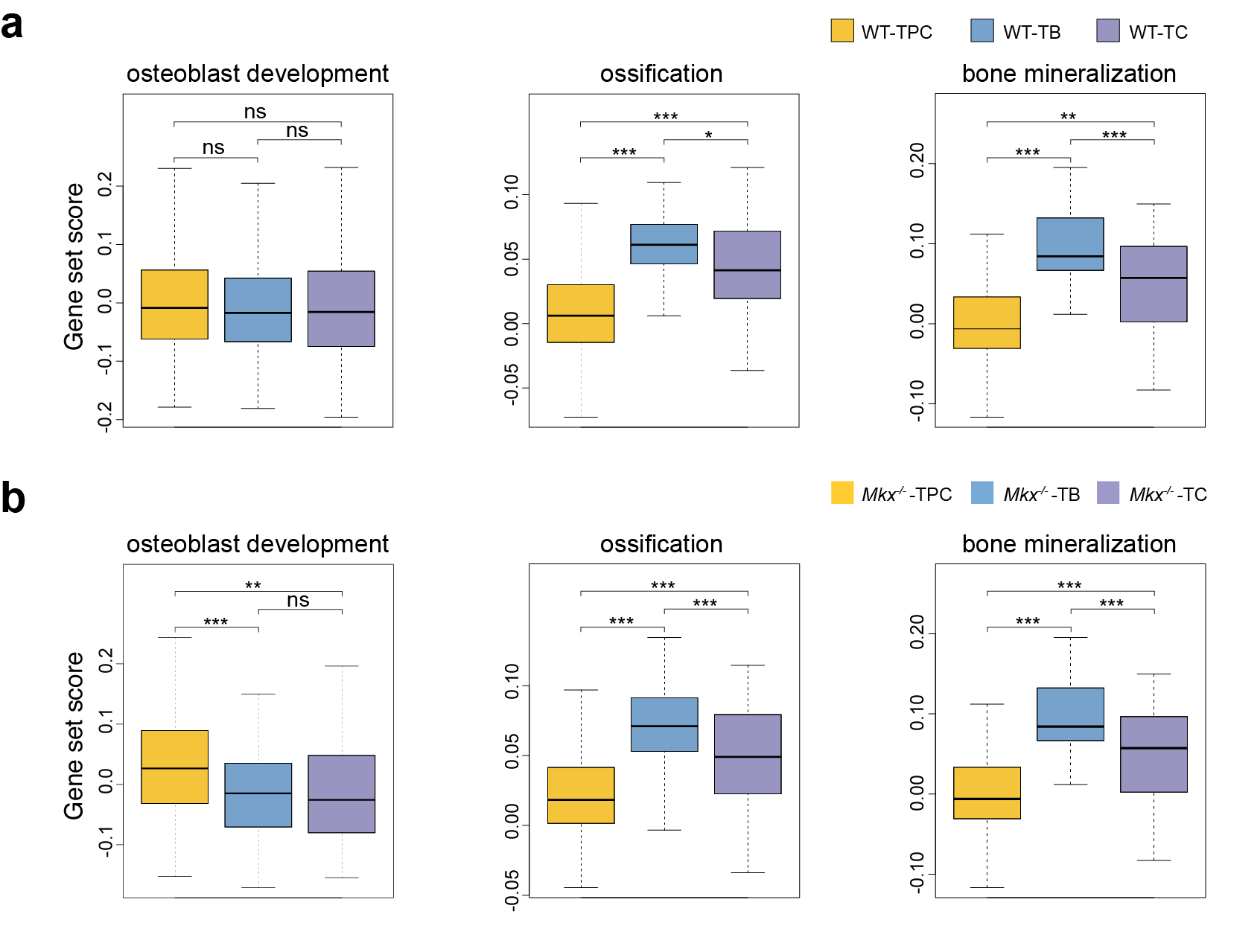


**Supplementary Fig. S4.** Gene set enrichment analysis of pathways related to osteoblast development, ossification, and bone mineralization in WT (**a**) and *Mkx^-/-^* (**b**) TPCs, TBs and TCs. ns: no significance; **p < 0.01; ***p < 0.001 (two-sided Wilcoxon rank-sum test). TPCs: tendon progenitor cells, TBs: tenoblasts; TCs: tenocytes.

**Supplementary Fig. S5**


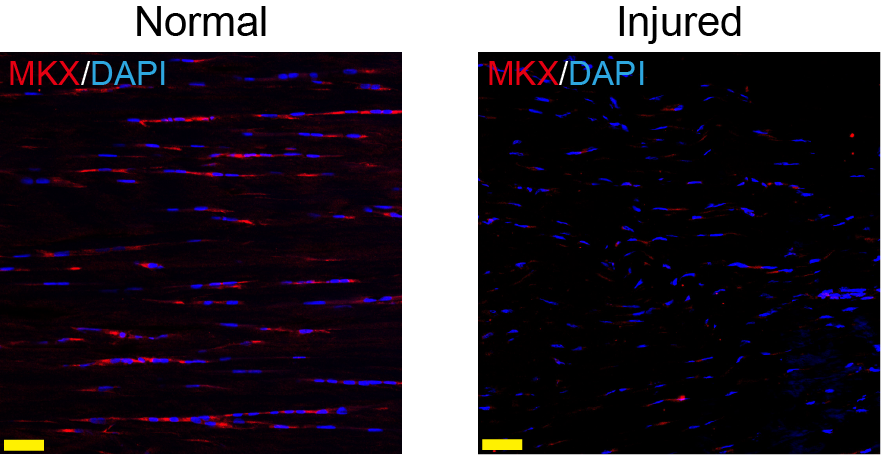


**Supplementary Fig. S5.** Immunofluorescence staining of Mkx in normal and injured rat tendons. Scale bar, 50 μm.

**Supplementary Fig. S6**


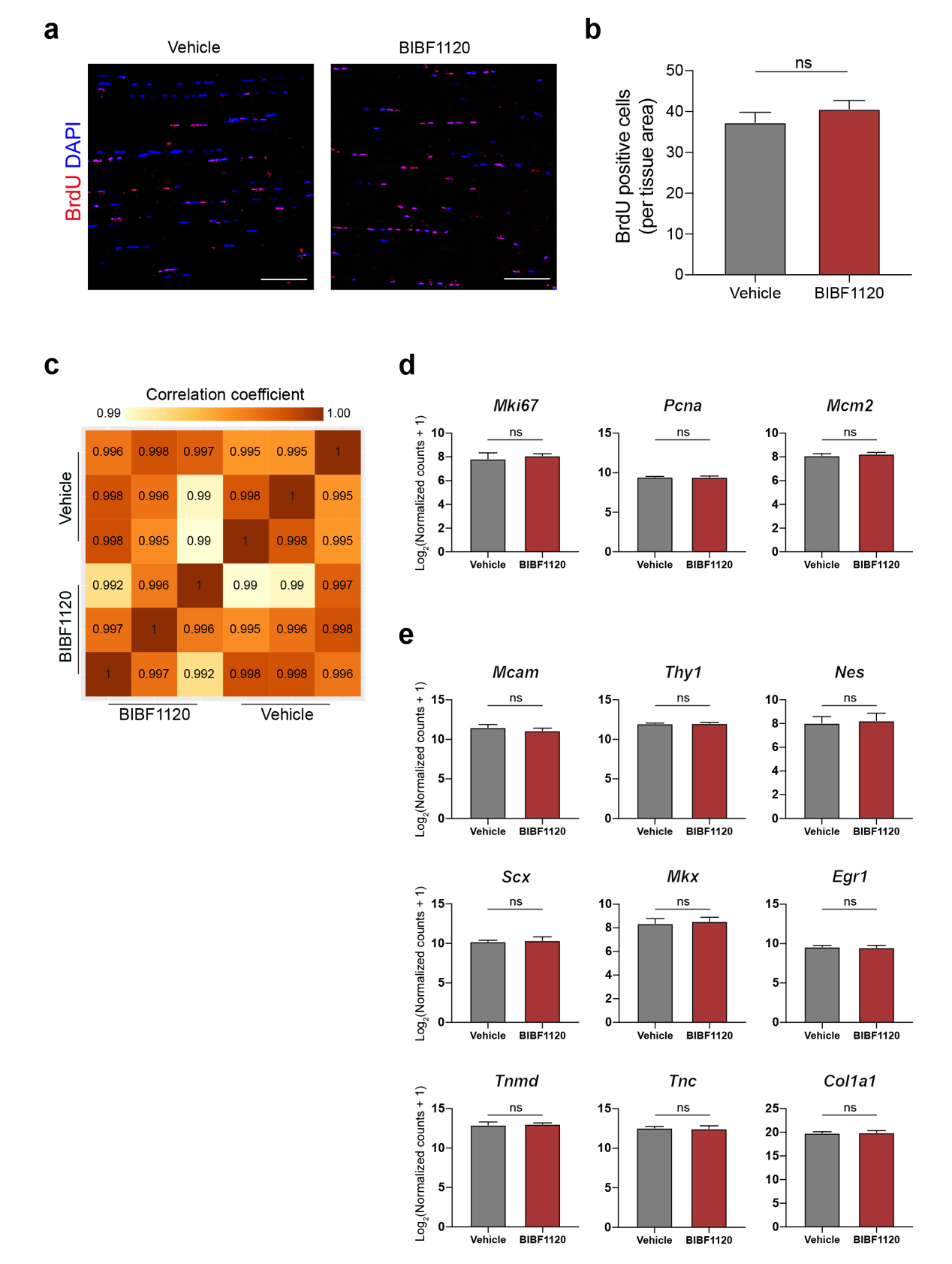


**Supplementary Fig. S6.** Effects of BIBF1120 on tendon cell proliferation and gene expression. **a, b** Immunostaining of BrdU (**a**) and statistical analysis of BrdU+ cells (**b**) treated with BIBF1120 or vehicle. The data are shown as the mean ± s.d. **c** Clustered heatmap showing the Pearson correlation coefficients of transcriptome-wide gene expression in tendons treated with BIBF1120 or vehicle. **d** Expression of proliferation markers in tendons treated with BIBF1120 or vehicle. **e** Expression of tendon and tendon progenitor/stem cell markers in tendons treated with BIBF1120 or vehicle. ns: no significance.
